# Mechanistic Research for the Student or Educator (Part II of II)

**DOI:** 10.3389/fphar.2022.741492

**Published:** 2022-07-12

**Authors:** Rehana K. Leak, James B. Schreiber

**Affiliations:** ^1^ Graduate School of Pharmaceutical Sciences, Duquesne University, Pittsburgh,, PA, United States; ^2^ School of Nursing Duquesne University, Pittsburgh, PA, United States

**Keywords:** mechanistic, hypothesis, physiology, biology, pharmaceutical, biomedicine, preclinical

## Abstract

This two-part series describes how to test hypotheses on molecular mechanisms that underlie biological phenomena, using preclinical drug testing as a simplified example. While pursuing drug testing in preclinical research, students will need to understand the limitations of descriptive as well as mechanistic studies. The former does not identify any causal links between two or more variables; it identifies the presence or absence of correlations. Parts I and II of this educational series encourage the student to 1) ensure the sensitivity and specificity of their measurements, 2) establish or optimize an appropriate disease model, 3) find pharmaceutical drug doses/concentrations that interfere with experimental disease processes, 4) leverage the literature and exploratory datasets to craft a mechanism-oriented hypothesis on drug binding and downstream effects, 5) and design a full-factorial experiment to test the hypothesis *after* sketching potential outcomes and imagining their interpretations. These creative goals facilitate the choice of the appropriate positive and negative controls to avoid false data interpretations. Here, Part II describes in detail how to test for a causal link between drug-induced activation of biological targets and therapeutic outcomes. Upon completion of this two-part series, the new student will have some of the tools in hand to design mechanistic studies, interpret the outcomes of their research, and avoid technical and theoretical pitfalls, which can otherwise decelerate scientific progress and squander human and financial resources.

## Introduction

Students and educators of the biomedical and pharmaceutical sciences need to understand how the physiological action of drugs on protein molecules is determined. To help meet this educational need, Part I of this series identifies solutions for common technical problems encountered while researching the biological impact of drug candidates. Here, Part II briefly describes how to test the hypothesis that a pharmaceutical drug candidate protects against a preclinical disease model by enhancing (or inhibiting) the physiological function of a specific protein in a cell culture-based assay. The protein is believed to act downstream of receptor binding by a drug candidate that prevents the toxicity of the experimental disease (see Figure 3C, Part I). Technical tools such as gene knockout, RNA interference, forced gene overexpression, and pharmacological antagonism or agonism can be leveraged to test the mechanistic hypothesis. As presented below, the process of designing the experiment for a full-factorial ANOVA forces the student to include critical control groups and mitigates the risk of false interpretations, in accordance with modern research guidelines described in Part I (Schlenker, 2016; Weissgerber et al., 2018; Percie du Sert et al., 2020a; Percie du Sert et al., 2020b; Garcia-Sifuentes and Maney, 2021).

### Problem 1: Formulating a Hypothesis

**FIGURE 1:**
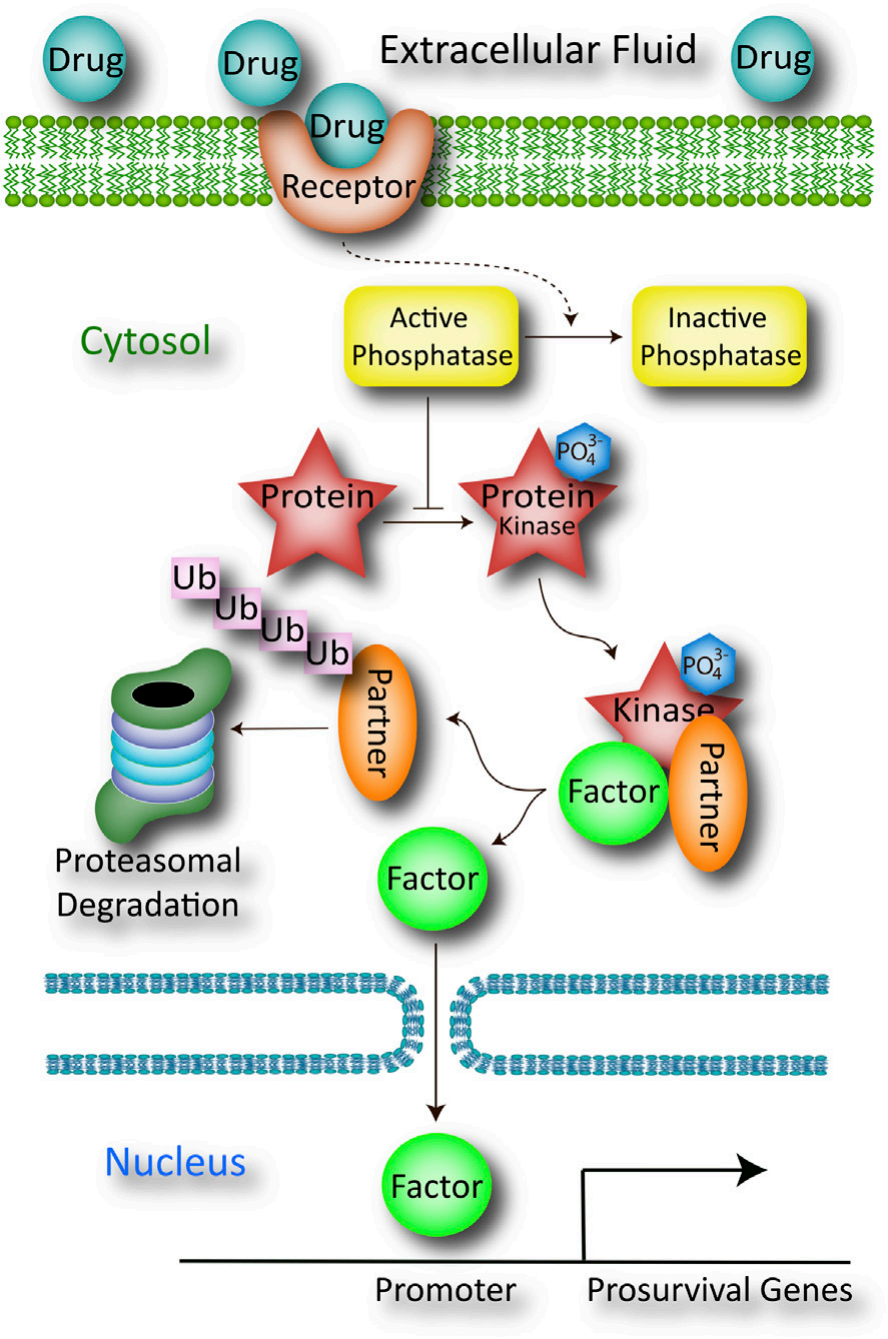
Schematic of a working hypothesis drawn from the literature or prior evidence collected using *in silico* computational modeling, *in vitro* cell culture work, *ex vivo* studies of organs or organ slices, *in vivo* studies in animals, or RNA sequencing and proteomics. The pharmaceutical drug candidate is believed to bind a plasma membrane-bound receptor (*e.g*., G-protein coupled receptor) that indirectly inactivates a cytosolic protein phosphatase through a series of G-protein mediated steps not shown here (dotted line). This inactivation step serves to increase the net phosphorylation state of the protein, shown as a red star with an attached PO_4_^3-^ group. The phosphorylated protein serves as a kinase that promotes the release of a transcription factor from its inhibitory partner through a second phosphorylation event. The detached inhibitory partner is rapidly ubiquitinated and degraded by the proteasome, and its absence permits the translocation of transcription factor across the nuclear membrane. Once the transcription factor has entered the nucleus, it binds the promoters of multiple genes involved in cellular defense. The products of those prosurvival genes then battle apoptosis to mitigate the toxic sequelae of the preclinical disease model.

Once a candidate biological pathway has been identified through a literature search, RNA sequencing, proteomics, or computational methods, it is helpful to sketch the working hypothesis in the larger context of the cell or tissue, as in Figure 1, although the “real life movie would be much more complex,” as quoted from Van Mil and colleagues (Mil et al., 2016). For sake of simplicity, the hypothesis will be tested in primary cultured cells harvested from genetically modified animals and studied *in vitro*, although the same arguments can be adapted to more complex *in vivo* approaches.

A key component of identifying the biological target of a drug candidate involves classic or forward pharmacology, often in combination with structure-activity relationships, to 1) optimize the binding of the target and activation/upregulation of downstream molecules, 2) reduce off-target effects, and 3) improve drug absorption, distribution, metabolism, and excretion (ADME). The ballooning cost of drug discovery, estimated to approximate a median of nearly $1B per drug candidate (Wouters et al., 2020), has fostered reliance on less-expensive *in silico* molecular modeling and *in vitro* cell culture techniques to partially meet these goals. As with all technologies, there are weaknesses of *in silico* and *in vitro* approaches, and they are not intended to fully replace animal work (Kubinyi, 2003). The pharmacokinetic and pharmacodynamic effects of the drug and its ADME properties must be examined *in situ* within the integrated context of the awake and behaving organism prior to clinical testing, including at the level of organ toxicity (Jobe et al., 1994; Ramaekers, 1998).

In the hypothetical example sketched in Figure 1, a comprehensive literature search suggests that the drug candidate directly binds a membrane receptor, and this target engagement sets in motion a cascade of downstream events that culminate in the inactivation of a cytosolic phosphatase (the dotted line indicates that the pathway is indirect). With phosphatase activity now suppressed, the drug indirectly boosts the phosphorylation and function of a protein (red star in Figure 1), which facilitates the dissociation of a transcription factor from its inhibitory partner, through as second phosphorylation event. When the inhibitory factor is removed from the kinase-associated complex, it is ubiquitinated and degraded by the proteasome. The unmoored transcription factor then translocates into the nucleus to promote the expression of prosurvival genes. The collective protein products of these genes improve mitochondrial generation of ATP, providing sufficient energy to repair damaged cells/tissues, thereby battling the experimental disease.

The direct binding of the drug to the membrane-bound receptor (often a G-protein coupled receptor) can be measured with traditional radioligand binding assays, but binding of the receptor does not guarantee the downstream effects shown in Figure 1. Rather, the binding assay would ideally be followed by measurements of drug-induced inactivation of the phosphatase, activation (phosphorylation) of the protein (*i.e.*, the red star), loss of expression of the inhibitory partner (and whether or not this loss is mitigated by proteasome inhibitors), translocation of the transcription factor from cytosol to nucleus, binding of the transcription factor to the expected promoters, and induction and expression of the correct set of prosurvival genes.

All the above events form various segments of the mechanism of action of the drug, even if the membrane receptor is the only direct target of the drug. For the sake of argument and brevity, we will proceed to test if the function of one protein (the red star) is *causally* linked to the positive impact of the drug candidate on the disease model.

**FIGURE 2:**
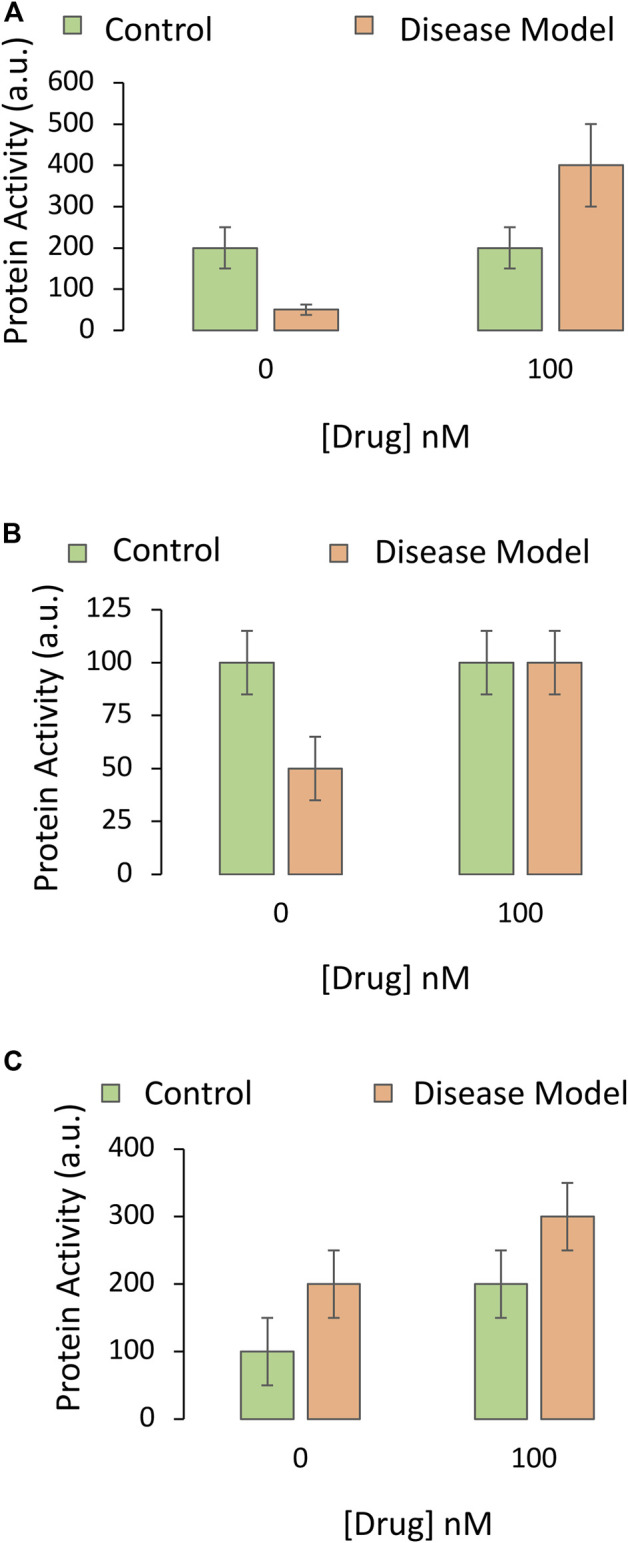
Impact of the drug candidate on the activity of a downstream target protein in an experimental disease model. A specific and sensitive assay is used to screen whether the pharmaceutical drug candidate increases the function of the protein hypothesized to mediate the protective impact of the drug against the experimental disease. The *Y* axis reflects protein activity levels (the dependent variable), and the *X* axis indicates one of the independent variables (application of vehicle or the most protective concentration of the drug, as determined in Figure 2 of Part I of this series). The orange and green bars refer to the second independent variable (the disease model or the non-diseased control, respectively). **(A)** The drug reverses the loss of protein activity elicited by the disease and causes an increase in this functional measure. **(B)** The drug abolishes the loss of protein activity elicited by the disease but does not increase this measure beyond baseline values. **(C)** The drug increases protein function in both the non-diseased and diseased groups. There is no statistical interaction between the drug and the disease, unlike in panels A and B

An idealized graph of the impact of the drug on the protein is depicted in Figure 2A; it shows that the disease model involves a loss of function of the protein, and that the drug not only prevents this drop, but increases the function of the protein under disease conditions. Even if there were no dramatic upregulation of the protein and the drug simply prevented loss of its function under disease conditions (Figure 2B), the protein may still be a suitable candidate for mechanistic follow-up work.

Another alternative is that the protein is upregulated as a compensatory response to the disease, and that the drug candidate simply increases baseline expression and activity of the protein, in the presence or absence of disease (Figure 2C). Although there is no statistical interaction between the impact of the drug and the disease in Figure 2C (see Part I), the drug need not be abandoned; the protein may still mediate beneficial effects of the drug by improving overall viability. On the other hand, if the drug also increases the activity of the protein under physiological (non-diseased) conditions, as shown by the second green bar in Figure 2C, it might have oncogenic potential or other off-target effects on healthy tissues.

Thus far, the activity of the protein is *correlated* with prevention of the disease, but the data do not reveal if upregulation of the protein by the drug candidate *causes* prevention of the disease. Even if the drug causes the entire series of events in Figure 1, none of the abovementioned measurements guarantee that these proteins form part of the cascade underlying the protective qualities of the drug. In other words, we have yet to test the following hypothesis:

Hypothesis 1: The drug candidate (or any kind of intervention) reduces the toxic effects of preclinical disease by upregulating the function of a protein.

Below, we guide the beginner scientist through various challenges and potential outcomes of testing this hypothesis with a simple primary cell culture-based viability assay.

### Problem 2: Deploying the Full-Factorial Three-Way ANOVA

To claim that a biological pathway is the means whereby the drug acts on the body, the researcher needs to modify its purported mechanism of action, to determine if this interference lessens the protective effects of the drug as expected. As stated above, this involves inhibiting the function of the protein with a number of tools, ranging from pharmacological antagonism of the protein to deletion of the gene that encodes the protein, or knocking down the translation of the protein from its mRNA. An advantage of employing a full-factorial three-way ANOVA to achieve this goal is that it forces inclusion of all the control groups, each run in parallel, in accordance with ARRIVE and other guidelines discussed in Part I (Schlenker, 2016; Weissgerber et al., 2018; Percie du Sert et al., 2020a; Percie du Sert et al., 2020b). For a comprehensive understanding of ANOVAs, the student should refer to additional sources (Ferguson and Takane, 1989; Schreiber).

**FIGURE 3:**
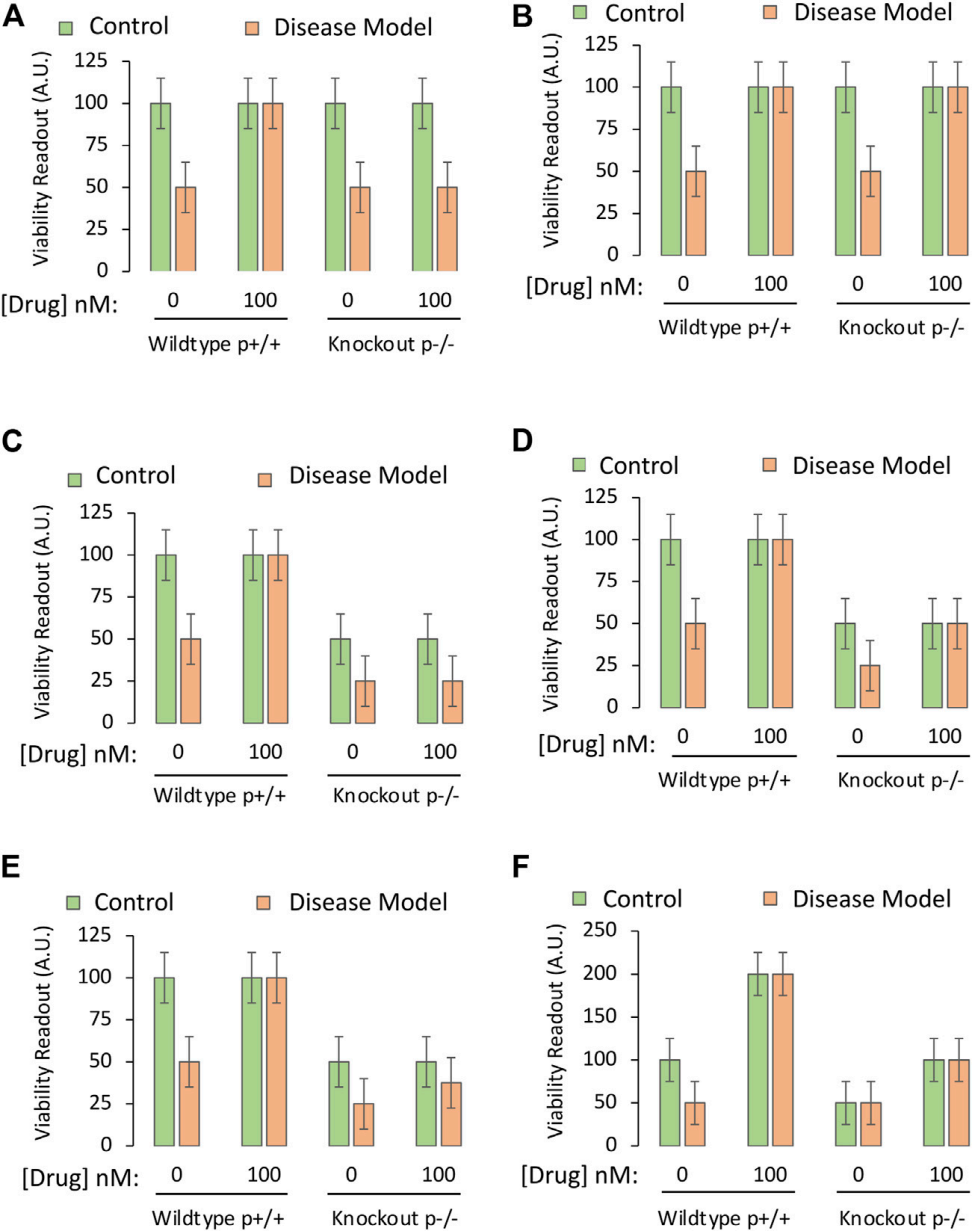
Testing the mechanistic hypothesis by leveraging knockout of the gene encoding a protein that is upregulated by the drug. A specific and sensitive cell culture-based viability assay is used to screen whether the drug candidate exerts its protective impact by engaging the protein of interest. The *Y* axes reflect cellular viability levels (the dependent variable), and the *X* axes indicate the first two independent variables: 1) Application of vehicle or the most protective concentration of the drug (as determined in Part I of this series), and 2) use of primary cells harvested from wildtype mice (cells with both alleles, labeled as p+/+) or homozygous mice with knockout of the gene encoding the protein (both alleles absent, labeled as p−/−). The orange and green bars refer to the third independent variable, the disease model or the non-diseased control, respectively. **(A)** The protective impact of the drug candidate is abolished when the gene coding for the protein is knocked out. **(B)** Knocking out the gene does not modify the protective impact of the drug candidate. **(C)** Knocking out the gene reduces basal viability under physiological conditions (green bars) and *also* abolishes the protective impact of the candidate drug under disease conditions (orange bars). **(D)** Knocking out the gene reduces basal viability under physiological conditions (green bars), but does not abolish, or even reduce, the protective impact of the candidate drug. **(E)** Knocking out the gene reduces baseline viability under physiological conditions and also partially dampens the protective impact of the candidate drug under disease conditions. **(F)**. Practice example for active learning; see text for details.

In our cell culture-based example, the three-way ANOVA allows us to test the impact of the following independent variables or factors on the dependent variable (Figure 3):

1) The disease state, of which there are two levels: 1) The non-diseased control or 2) a stimulus that induces the experimental disease *in vitro* with cell loss at LC_50_ values (see Part I about controlling for the effects of the vehicle that the disease-inducing stimulus is dissolved in).
• This independent variable (disease state) is plotted as green versus orange bars.
2) Treatment, of which there are two levels: 1) Vehicle or ii) the most effective, nontoxic concentration of the drug (100 nM)
• The independent variable of treatment status is plotted twice on the *X* axis.
• Inclusion of multiple concentrations of the drug may not be economical or necessary, if the full-fledged concentration-effect study for this project has already been completed (see Part I of this two-part series). Exceptions are discussed below.
3) Protein expression, of which there are two levels: 1) Normal expression in control cells harvested from wildtype mice, or 2) lack of expression in cells harvested from genetically modified mice with knockout of the gene.
• This independent variable (*i.e.,* the presence or absence of the gene encoding the protein) is plotted once on the *X* axis, as demarcated with the horizontal black lines.

### Problem 3: Technical Confounds in Mechanistic Research

In general, pharmacological antagonists are less selective than targeted genetic approaches, but it is helpful to use both pharmacologic and genetic editing to show that two different complementary approaches yield the same outcome. If gene deletion is lethal to the embryo in animal studies or lethal to cells cultured *in vitro*, the researcher can still use pharmacological antagonists or knockdown techniques. Even if the fetus survives the complete absence of the protein, there might be serious developmental deficits that affect baseline levels of the measurement outcomes. Other potential confounds of embryonic knockout of proteins include the compensatory, developmental upregulation of other proteins with similar functions, which can result in a net lack of change in measurement outcomes (Barbaric et al., 2007). Such compensatory changes can lead to the erroneous conclusion that the protein is irrelevant for survival when the candidate drug is administered (*i.e.,* a false negative). Under those circumstances, it might be wiser to employ a pharmacological antagonist.

There can be other confounding factors when employing gene knockout techniques, including off-target effects of gene deletion methods such as CRISPR-Cas9 (Zhang et al., 2015). Furthermore, if the researcher only studies the fraction of cells that manage to survive deletion of the gene, the remaining cells under scrutiny *in vitro* or *in vivo* might not be representative of the original cellular population. This can pose a problem during long-term *in vitro* selection of only a few genetically modified cells.

One alternative is to knock down (but not abolish) the translation of the protein through RNA interference in adulthood, perhaps through a virus (*e.g.,* lentivirus, adeno-associated virus, *etc.*) added directly to the target tissue or cells, electroporation, or non-viral nucleic acid delivery systems (*e.g.*, transfection “helpers” such as cationic lipid formulations or nanoparticles) (Grimm et al., 2005; Veldhoen et al., 2008; Dreyer, 2011). However, the infectivity rate and/or efficiency of the knockdown are sometimes impractically low and the knockdown method can be associated with toxicity and off-target effects, such as inflammation (Guo et al., 2019; Li et al., 2019).

A second alternative is to use a conditional *in vivo* knockout model, in which, for example, delivery of tamoxifen in adulthood stimulates deletion of a gene flanked by two inserted *loxP* sites, within cells that express the DNA recombinase Cre (Friedel et al., 2011). The latter approach avoids the potential developmental changes that might compensate against embryonic knockout of a protein and can also be used to target specific cell types (Smith et al., 2020). For pitfalls of the Cre-lox system, see the discussion by Song and Palmiter (Song and Palmiter, 2018). The student will also need to include control groups to avoid confounding actions of tamoxifen, which will bind the estrogen receptor throughout the body after systemic routes of administration (Goodsell, 2002).

### Problem 4: Putting the Mechanistic Hypothesis to the Test

Idealized data from successful mechanistic tests are illustrated in Figure 3A, via the cell-based viability assay described in Part I of this series. When assessing cells with deletion of the gene that encodes the protein of interest, there is total loss of efficacy of the drug—it fails to improve viability under diseased conditions because the protein cannot be engaged. The investigator would then accept the test hypothesis, provided they have ensured that the gene is indeed deleted by testing mRNA and protein expression (see Part I).

If the absence of the protein does not mitigate the protective properties of the drug (Figure 3B), the researcher would reject the test hypothesis and conclude that the drug protects against the disease by affecting molecules *other* than the protein in question.

As the hypothetical target is assumed to serve as a “prosurvival” protein, its absence might decrease basal viability, as in Figure 3C. Here, the protein exerts a positive impact upon basal viability and *also* mediates the effects of the drug candidate. The test hypothesis would be accepted.

In Figure 3D, the protein is still a prosurvival protein, without which basal viability is lowered, but it does not mediate the protective effects of the candidate drug, as its absence has no impact on the latter measure. In Figures 3B,D, targets other than this protein mediate the protective effects of drug D, and the researcher might have to consult their RNA sequencing or proteomic data sets again.

If the protein only partially mediates the protective impact of the drug, the results may appear as in Figure 3E, where loss of protein expression reduces, but does not completely abolish the protective effects of the drug. In this event, the drug prevents the toxic consequences of experimental disease, at least partly, through the protein in question.

As an active learning exercise, the student should reason through some of their interpretations of Figure 3F, before reading the arguments presented below.

A number of possible explanations for data displayed in Figure 3F are presented below, where we will systematically work through the green and orange bars, from left to right:

1. Assume that the disease model reduces the function of the prosurvival protein to near zero in half of the cells present, and thereby kills this vulnerable half, only leaving cells behind that do not express the protein, but survive by means of *other* prosurvival proteins (first two bars in Figure 3F).
2. Next, assume that the drug prevents the disease model from killing half the cellular population because it dramatically increases the function of those other prosurvival proteins, and their upregulation drives cell division to the maximal levels that the plate size and media nutrients can support (second set of bars in Figure 3F). These alternative prosurvival pathways overwhelm any toxic impact of disease, and the viability readout is doubled in both the green and orange bars when the drug is applied.
3. In the third set of bars, the protein is completely absent, and, therefore, half the cells no longer survive. Here, the disease model exerts *no additional impact* because there is no protein to inhibit, leaving the investigator with a viability readout of 50 (in arbitrary units) for both the green and orange bars (absence or presence of disease, respectively).
4. In the fourth set of bars, the drug improves viability by upregulating the function of the *other* prosurvival proteins, even if it fails to do so to maximal levels. The observation that viability is doubled by the drug—even in the absence of the protein in question and in the absence of disease—informs the investigator that the drug protects against loss of *basal* viability but does not engage the knocked-out protein to elicit this effect.
5. The hypothesis states that upregulation of the protein is the mechanism of action of the drug *under diseased conditions.* The third set of bars in Figure 3F reveal that the disease no longer has any toxic effect when the protein is absent. It is not possible to test the hypothesis that the drug protects against the toxicity of a disease, when the disease cannot even be provoked.
6. The researcher is forced to try other means to test the hypothesis, as the data are inconclusive. The researcher might employ RNA interference-mediated knockdown of the protein rather than total deletion of the gene, or concentrations of an antagonist that decrease the function of the protein, but *not to the fullest extent*, such that a reduction in viability under disease conditions could still be resolved in the third set of bars.

### Problem 5: Complementary Use of Pharmacological and Genetic Approaches

Point number 6 in the above list highlights the importance of reporting concentration-effect curves, a major gap in the modern reductionistic approach to biomedical science (Mullane et al., 2014) and a caveat to the binary gene-deletion approach employed in Figure 3. Concentration-dependent inhibition of protein function is more readily achieved with a pharmacological antagonist. If the latter route is preferred, one option to defend against confounding off-target effects is to use two antagonists that are in different drug classes and downregulate the function of the same protein through distinct mechanisms; the statistical probability that their off-target effects also overlap (as much as their effects on the protein itself) is low. Simply put, if two independent classes of antagonists abolish or mitigate the impact of the drug, it is reasonable to conclude that the drug works fully or partially through upregulation of the protein in question.

**FIGURE 4:**
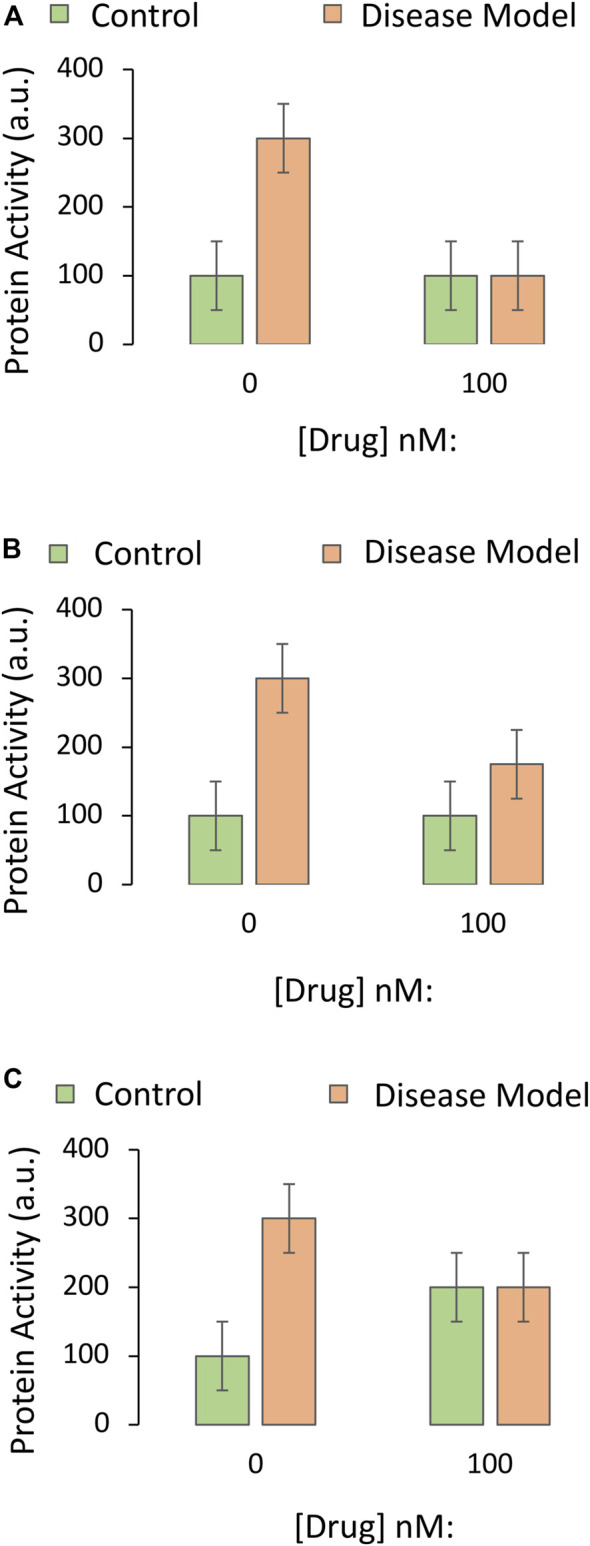
Impact of the drug candidate on the activity of a downstream target protein in an experimental disease model. A specific and sensitive assay is used to screen whether the drug candidate decreases the function of a pro-apoptotic protein. The *Y* axes reflect pro-apoptotic protein activity levels (the dependent variable), and the *X* axes indicate the first independent variable (application of vehicle or the most protective concentration of the drug, as determined in Part I of this series). The orange and green bars refer to the second independent variable (the disease model or the non-diseased control, respectively). **(A)** The drug candidate abolishes the increase in pro-apoptotic protein activity that is elicited by the disease. **(B)** The drug reduces but does not fully prevent the increase in pro-apoptotic protein activity elicited by the disease. **(C)** The drug candidate decreases pro-apoptotic protein function in diseased groups, but the data raise concerns about off-target, toxic effects of the drug in non-diseased, control tissues.

Rather than upregulating prosurvival proteins, the drug in our example might protect against disease by reducing the function of a pro-apoptotic protein. In this scenario, using knockout cells, antagonists, or protein knockdown is less straightforward. Possible outcomes of the protein assay are illustrated in Figure 4. If the disease model is toxic due to an upregulation of a pro-apoptotic protein, the drug might be expected to prevent the disease-induced increase in protein function (Figure 4A) or to lower the size of the increase (Figure 4B). Inclusion of all the controls for the two-way ANOVA forces the investigator to examine the impact of the drug on the protein in normal, non-diseased tissue (second green bar). If the drug encourages a rise in the function of a pro-apoptotic protein in the non-diseased control group (Figure 4C), this reveals its potential hazards in healthy cells or tissues.

Rather than employing cells with knockout of the gene, forced *overexpression* of a transgene encoding the protein may allow researchers to directly interfere with the *downregulation* of the protein by a candidate drug. Alternatively, a pharmacological agonist could be employed. First, the student researcher should ensure that overexpression of the gene does increase the levels and function of the protein it encodes (see Part I).

**FIGURE 5:**
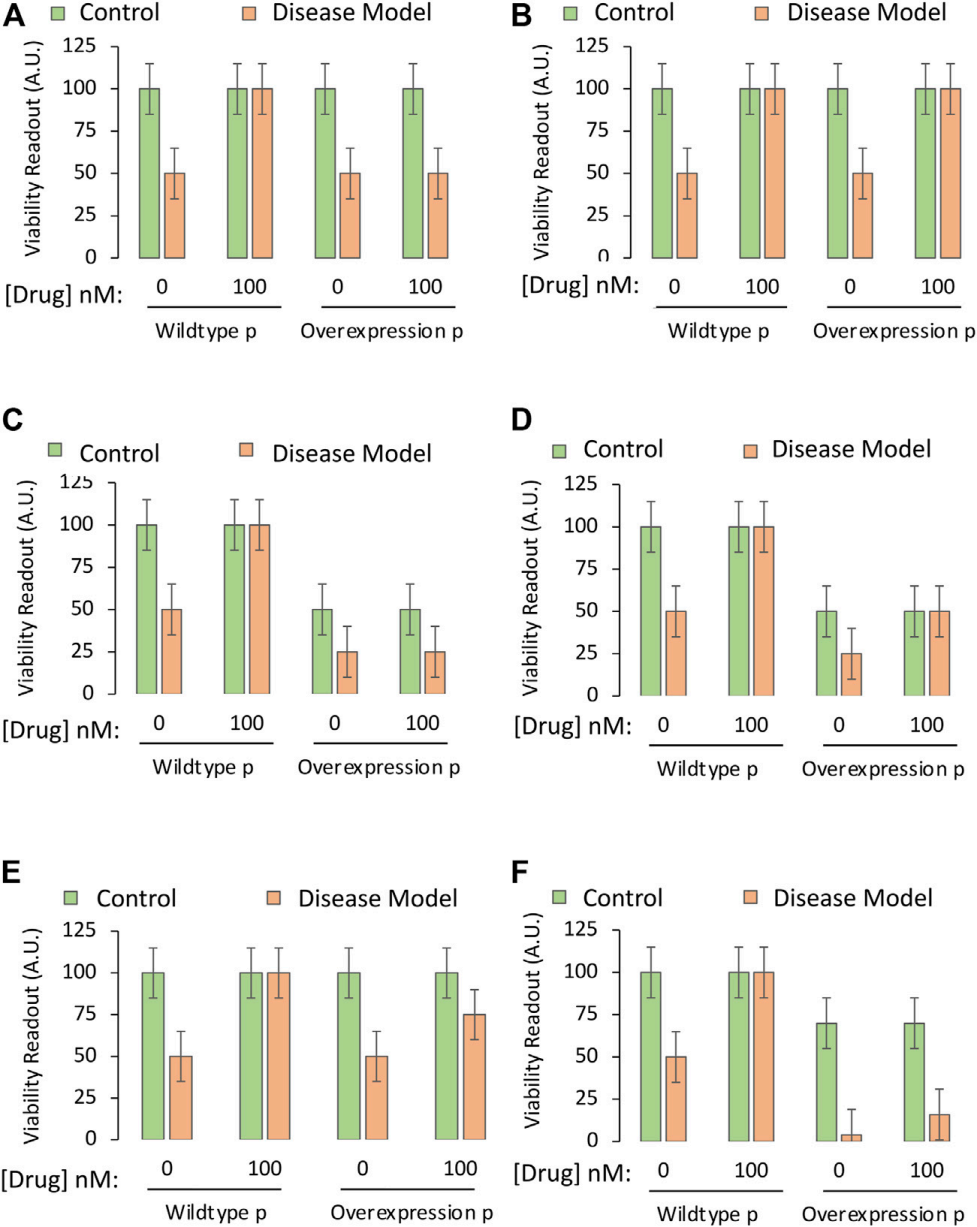
Testing the mechanistic hypothesis by leveraging overexpression of the gene encoding a protein that is downregulated by the drug. A specific and sensitive cell culture-based viability assay is used to screen whether the drug candidate exerts its protective impact through suppression of the function of the pro-apoptotic protein. The *Y* axes reflect cellular viability levels (the dependent variable), and the *X* axes indicate two independent variables: 1) Application of vehicle or the most protective concentration of the drug (as determined in Part I of this series), and 2) use of cells harvested from wildtype mice or genetically modified mice that overexpress the gene coding for the pro-apoptotic protein, labeled “p”. The orange and green bars refer to the third independent variable, the disease model or the non-diseased control, respectively. **(A)** The protective impact of the drug candidate is abolished when the pro-apoptotic protein is overexpressed. **(B)** Overexpression of the pro-apoptotic protein does not reduce the protective impact of the drug as hypothesized. **(C)** Overexpression of the gene reduces basal viability under physiological conditions and also abolishes the protective impact of the drug. **(D)** Overexpression of the gene reduces baseline viability under physiological conditions, but does not modify the protective impact of the drug. **(E)** Overexpression of the gene partially reduces the protective impact of the drug candidate. **(F)** Overexpression of the gene reduces basal viability under physiological conditions, but also synergizes with disease conditions to cause massive cell death, rendering it impractical to test the mechanistic hypothesis.

Simplified data that allow acceptance or rejection of the test hypothesis are displayed in Figures 5A,B, respectively. As the protein in this example is pro-apoptotic, its overexpression might also decrease basal viability, but the overexpression approach still permits acceptance or rejection of the test hypothesis in Figures 5C,D, respectively. If downregulation of the pro-apoptotic protein is only partially responsible for the protective effects of the drug, the data might appear as in Figure 5E, where overexpression of the protein does not fully abolish the protective effects of the drug, and the test hypothesis is accepted.

If the protein is strongly pro-apoptotic and its overexpression is overly toxic to cells when it synergizes with disease processes, as in Figure 5F (third orange bar), it might be too difficult to protect cells with the candidate drug. For example, severely stressed cells might yield to necrotic types of injury that are impossible to reverse even at early stages. Furthermore, even if there is a small increase in viability with the candidate drug in the fourth orange bar of Figure 5F, the viability values of the last two orange bars might be too close to background levels, and out of the dynamic range of the assay (see Part I).

Many investigators do not employ the overexpression approach to test whether their drug is protective by downregulating the protein, but employ knockout cells or animals. The rationale behind the knockout approach in this scenario is that the drug should have no *additional* protective properties in knockout cells compared to wildtype cells—if the drug does rely on reducing the function of the protein in question.

**FIGURE 6:**
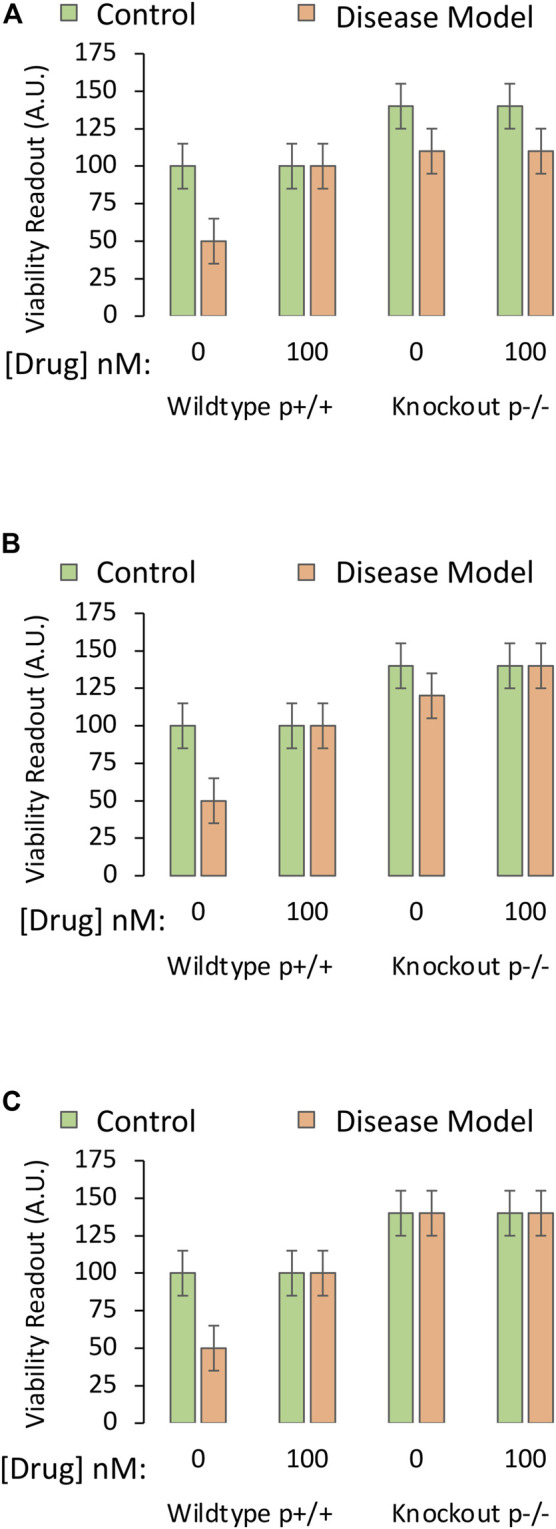
Testing the mechanistic hypothesis by leveraging knockout of the gene encoding a protein that is downregulated by the drug. A specific and sensitive cell culture-based viability assay is used to screen whether the drug candidate exerts its protective impact through suppression of the function of the pro-apoptotic protein. The *Y* axes reflect cellular viability levels (the dependent variable), and the *X* axes indicate two independent variables: 1) Application of vehicle or the most protective concentration of the drug (as determined in Part I of this series), and 2) use of cells harvested from wildtype mice or knockout mice with deletion of the gene coding for the pro-apoptotic protein, labeled “p”. The orange and green bars refer to the third independent variable, the disease model or the non-diseased control, respectively. **(A)** Deletion of the gene that codes for pro-apoptotic protein P increases basal viability, mitigates the toxicity of the experimental disease, and abolishes the protective impact of the drug candidate. **(B)** Deletion of the pro-apoptotic gene increases basal viability, mitigates the toxicity of the disease, but does not abolish the protective impact of the drug. **(C)** Deletion of the pro-apoptotic gene increases basal viability but also abolishes the toxicity of the disease model. Without this pro-apoptotic gene, the impact of the disease is not evident, and the mechanistic hypothesis is untestable.

If a pro-death protein partly responsible for induction of the disease is knocked out, one might expect less toxicity when the disease is experimentally induced, as well as an increase in basal viability (Figure 6A). In this scenario, the drug cannot prevent the partial toxicity of the disease under knockout conditions, if it relies on *further* downregulating the protein (as the protein is already gone), and the investigator would accept the test hypothesis. If, however, the drug is able to fully improve viability under diseased, knockout conditions (last orange bar Figure 6B), the drug cannot be relying on changing a protein that is absent, and the test hypothesis would be rejected.

### Problem 6: Interpreting the Unexpected

If removal of a pro-apoptotic protein *completely* abolishes the capacity of the disease to induce cell death, the researcher would not be able to determine if the drug has *additional* protective effects or not, as there is no loss of cells in the third set of bars and, therefore, no toxicity to prevent (Figure 6C). In this case, the investigator cannot reject or accept the test hypothesis. As argued above, an antagonist against the protein could be employed, to achieve some reduction in viability under disease conditions. This approach would allow the researcher to determine if the drug continues to mitigate the disease, despite interference with the function of the protein.

## Conclusion

To summarize, the mechanism of action of a pharmaceutical drug candidate is identified with pharmacological or genetic approaches that modify the expression/activity of the drug targets. Without interference with the proposed mechanism of action, a causal link between activation (or inhibition) of the biological target and the therapeutic outcomes of drug treatment cannot be established. Planning for a full-factorial ANOVA forces the student to include the appropriate control groups and mitigates the risk of false positive or false negative interpretations of scientific findings. There are, of course, many more complicated examples of real-life data than depicted here, including in descriptive work. For a real-life example of controls for edifying full-factorial analyses, the student can refer to a descriptive report showing an unexpected lack of lasting therapeutic effects of a drug (and evidence of some toxicity) in an animal model of acute dopaminergic neuron loss (Nouraei et al., 2016). Although there are caveats of this study (see its Discussion section), the inclusion of all control groups in full-factorial ANOVAs mitigated the risk of Type I and II errors (false positive and false negative interpretations, respectively). Finally, the student is also encouraged to think beyond current reductionist approaches (Jobe et al., 1994; Winquist et al., 2014) and view biomedical research as an integrative whole that spans molecular and systems biology.

## Data Availability

The original contributions presented in the study are included in the article/Supplementary Material, further inquiries can be directed to the corresponding author.
